# CTNNB1 in neurodevelopmental disorders

**DOI:** 10.3389/fpsyt.2023.1143328

**Published:** 2023-03-16

**Authors:** Wenting Zhuang, Tong Ye, Wei Wang, Weihong Song, Tao Tan

**Affiliations:** ^1^Oujiang Laboratory (Zhejiang Lab for Regenerative Medicine, Vision and Brain Health), Key Laboratory of Alzheimer's Disease of Zhejiang Province, Institute of Aging, Wenzhou Medical University, Wenzhou, China; ^2^Department of Neuroscience, Baylor College of Medicine, Houston, TX, United States

**Keywords:** CTNNB1, **β**-catenin, neurodevelopmental disorders, intellectual disability, autism spectrum disorder, schizophrenia

## Abstract

CTNNB1 is the gene that encodes β-catenin which acts as a key player in the Wnt signaling pathway and regulates cellular homeostasis. Most CTNNB1-related studies have been mainly focused on its role in cancer. Recently, CTNNB1 has also been found involved in neurodevelopmental disorders (NDDs), such as intellectual disability, autism, and schizophrenia. Mutations of CTNNB1 lead to the dysfunction of the Wnt signaling pathway that regulates gene transcription and further disturbs synaptic plasticity, neuronal apoptosis, and neurogenesis. In this review, we discuss a wide range of aspects of CTNNB1 and its physiological and pathological functions in the brain. We also provide an overview of the most recent research regarding CTNNB1 expression and its function in NDDs. We propose that CTNNB1 would be one of the top high-risk genes for NDDs. It could also be a potential therapeutic target for the treatment of NDDs.

## 1. Introduction

In humans, CTNNB1 (transcript number: NM_001904) is located at chromosome 3q 22.1 (chr3:41240942–41281939). Its typical transcript contains 14 protein-coding exons, which encode β-catenin consisting of 781 amino acids (1). In mice, Ctnnb1 is located at chromosome 9. β-catenin is a key component of the complex that mediates intercellular adhesion and is a critical downstream molecule of the canonical Wnt signaling pathway (2). CTNNB1 was initially described as a potential tumor suppressor gene as somatic Ctnnb1 deletion or mutation, which is accompanied by loss of gene expression, is linked to tumor development in a variety of cancers (3). Further studies also suggest its role in the CNS, especially in cortical development (4), neural stem cell proliferation and neurogenesis (5), neuronal apoptosis (6), cell adhesion (7), and synaptic connection (8).

Neurodevelopmental disorders (NDDs) are a class of highly heritable and heterogeneous disorders caused by defects during early brain development, including autism spectrum disorder (ASD), intellectual disability (ID), schizophrenia (SCZ), and epilepsy (9). The prevalence of ID is about 1% in the UK as reported in 2017 (10). And 1 in 44 children were diagnosed with ASD in the United States in 2021 (11). Although it occurs during childhood, the clinical manifestations of NDDs will last for life, which causes a serious socioeconomic burden. Risk factors of NDDs include genetic, epigenetic, and environmental factors, among which the genetic factors play an important role in the pathogenesis of NDDs (12). In recent years, abundant evidence has been accumulated to draw the causal relationship between CTNNB1 and NDDs (13).

In this review, we discuss a wide range of aspects of CTNNB1 and its physiological and pathological functions in the brain. We also provide an overview of the most recent research regarding CTNNB1 expression and its function in neurodevelopmental disorders.

## 2. CTNNB1 and Wnt/**β**-catenin signaling pathway

**Figure 1:**
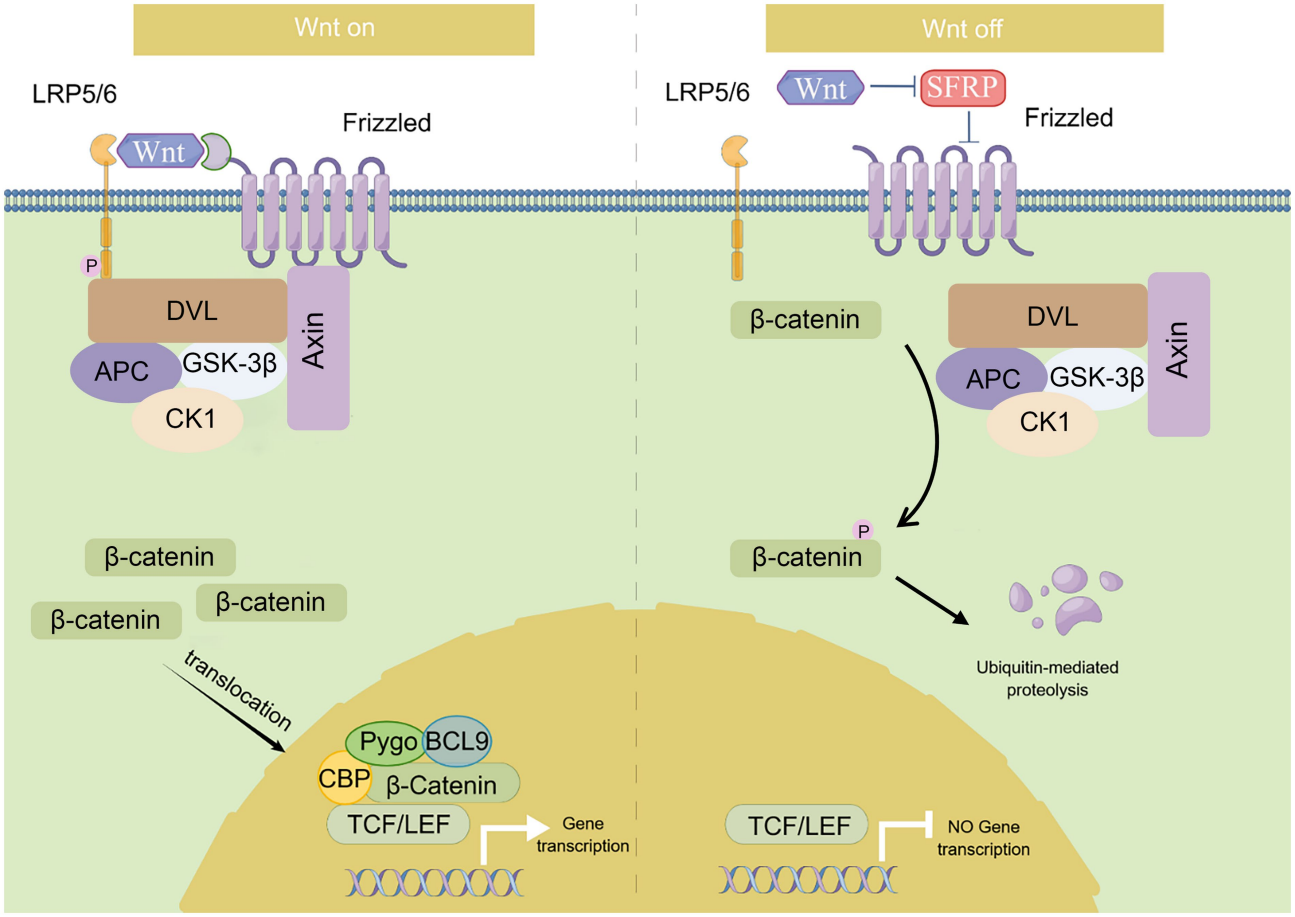
Summary of Wnt/β-catenin signaling pathway. This diagram depicts the canonical Wnt/β-catenin signaling pathway. Wnt/β-catenin signaling is activated (on-state) in the presence of Wnt ligand, which binds to Frizzled receptor and LRP5/6, and recruits Dishevelled (DVL) which restrains the destruction complex (GSK-3β, CK1, Axin, and APC) to formulate. β-catenin is accumulated in the cytoplasm and translocates into the nucleus where it facilitates gene transcription as a co-activator of LEF1/TCFs. While in the absence of the Wnt ligand (off-state), this signaling pathway is inactivated. β-catenin is phosphorylated by the destruction complex and then ubiquitinated by the SCF (Skp1/cullin/F-box) complex for proteasome degradation.

CTNNB1 encodes β-catenin which is a key player in the canonical Wnt/β-catenin signaling pathway. This pathway is highly conserved and plays a broad role in almost all tissues (14). The Wnt/β-catenin signaling pathway is activated (on-state, left panel of Figure 1) when Wnt binds to the receptor complex composed of 7-transmembrane helix Frizzled and low-density lipoprotein receptor-related proteins (LRP5/6) (15). It phosphorylates LRP6 and recruits Dishevelled (DVL), a proteins serve as a pivotal mediator of Wnt/β-catenin signaling (16), that restrains the destruction complex (GSK-3β, CKIα, Axin, and APC) to formulate. Following this, the destruction complex attaches itself to the cell surface and inhibits the phosphorylation of β-catenin, thereby preventing its ubiquitination. And the stabilized cytoplasmic β-catenin then translocate into the nucleus, where it works with the transcription factors, particularly TCF and LEF, to control the transcription of target genes (17).

This pathway is inactivated (off-state, right panel for Figure 1) when Wnt is absent. The cytoplasmic destruction complex transports β-catenin to the proteasome for degradation (18). In the absence of β-catenin, the activity of the TCF/LEF transcription factor is suppressed in the nucleus. This pathway is primarily involved in regulating cell differentiation, proliferation, and inflammation (19–21). Early developmental studies in mice have also shown that Ctnnb1 regulates the Wnt signaling pathway at the transcriptional level in preimplantation embryonic development and gastrulation (22).

In addition to its role in the Wnt/β-catenin signaling pathway, β-catenin is also a crucial component in the formation and maintenance of adherens junctions (AJs). AJs are specific types of cell–cell junctions that provide tissue stability by binding to the cytoskeleton and forming extracellular bonds with neighboring cells (23, 24). They are essential for morphogenesis and remodeling of tissues and organs. AJs also play a significant role in the cortical development (25) and maintenance of the neuroepithelium’s structural integrity (26), particularly in the early telencephalon, where cell–cell adhesion is mediated by β-catenin. Moreover, β-catenin is also known to involve in cadherin-mediated adhesion. As a key component of cadherin adhesion complexes, β-catenin functions as a scaffolding protein that stabilizes the cadherin adhesion complex and anchors it to the actin cytoskeleton, thus promoting the formation of strong cell–cell contacts (26). The adhesion complex is present in both pre- and postsynaptic regions and has a significant impact on the modulation of synapse formation and synaptic plasticity (27).

## 3. CTNNB1 transcription in the central nervous system

**Figure 2:**
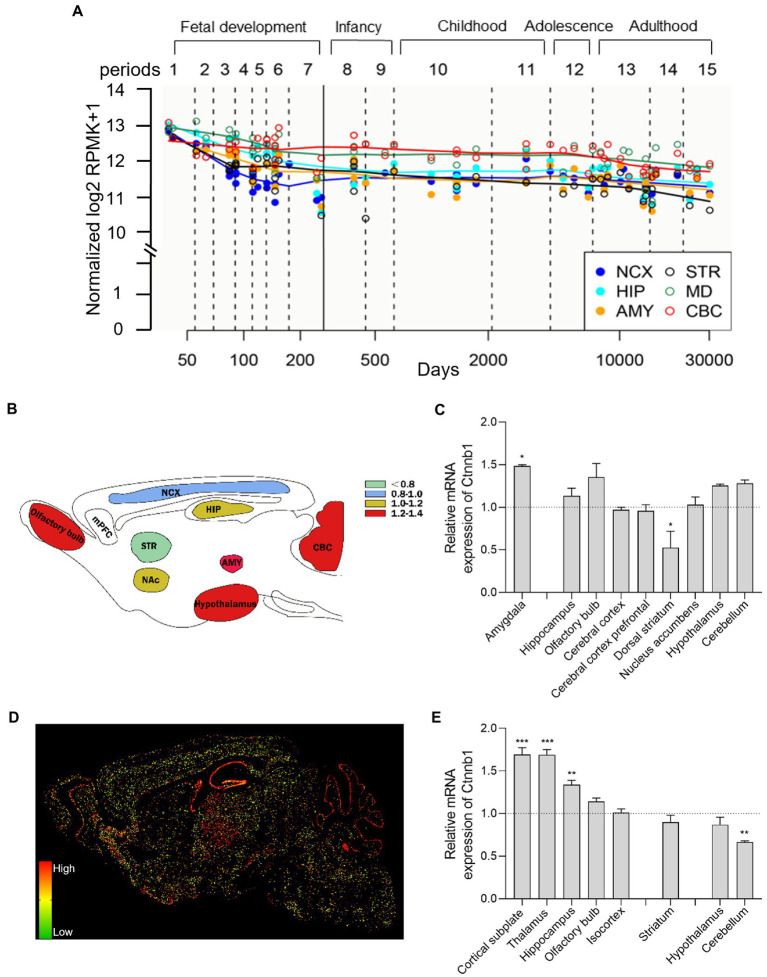
Brain-wide CTNNB1 expression and distribution. **(A)** Trajectory plot showing the expression of CTNNB1 during fetal development (period 1–7), early stages (period 8–9), youth (period 10–11), puberty (period 12), and adulthood (period 13–15) from different brain regions in human: NCX (the neocortex), HIP (hippocampus), AMY (amygdala), STR (striatum), MD (mediodorsal nucleus of the thalamus), and CBC (cerebellar cortex). Reprinted and modified from Human Brain Transcriptome dataset (https://hbatlas.org/, search gene Ctnnb1). **(B,C)** Schematic illustration and bar graph of Ctnnb1 expression in different brain regions of mice by using high-throughput gene expression profiling. Data obtained from BioGPS (http://biogps.org/#goto=welcome, search gene Ctnnb1). **(D,E)** The sagittal section and bar graph of the *in-situ* hybridization of Ctnnb1 mRNA signals in mice, obtained from Allen Institute ISH data (https://mouse.brain-map.org/, experiment 196, 197, 79904258). One–Way ANOVA with Holm-Sidak’s multiple comparisons test compared to whole-brain average expression, ***p < 0.001, **p < 0.01, *p < 0.05.

CTNNB1 is widely expressed in multiple tissues and organs and is highly expressed in the brain, uterus, lung, bladder, and kidney et al. (28). In the adult primate brain, CTNNB1 is highly expressed in the gray matter of the dorsolateral prefrontal cortex (DLPFC) and hippocampus (29, 30). To evaluate the comparative temporospatial transcription of CTNNB1 in the human central nervous system (CNS), we searched the Human Brain Transcriptome (HBT) dataset (https://hbatlas.org/, search gene Ctnnb1), which is based on Affymetrix GeneChip arrays (31). As showing in Figure 2A, CTNNB1 transcription is comparable and uniform in multiple brain regions, including the neocortex (NCX), hippocampus (HIP), mediodorsal nucleus of the thalamus (MD), and cerebellar cortex (CBC). Relatively higher CTNNB1 transcription level was found in all brain regions during the early embryonic development stage. During fetal development, the expression of CTNNB1 decreased constantly. But in NCX and CBC, it increased gradually from late embryonic to infancy and remained stable from childhood to adulthood. We also noticed higher CTNNB1 transcription in CBC and MD, and lower expression in the amygdala (AMY), and striatum (STR), compared to other brain regions, which persisted from late embryonic throughout adulthood.

To explore the brain-wide Ctnnb1 transcription in mice, we searched the high-throughput gene expression profiling of mouse brains from bioGPS (http://biogps.org/#goto=welcome, search gene Ctnnb1). As shown in Figures 2B,C, Ctnnb1 is relatively highly expressed in the amygdala (fear condition and aggression (32)), cerebellum (responsible for balance and movement (33)), hypothalamus, and hippocampus (spatial memory and learning (34)), but below average expression level was found in the dorsal striatum (amygdala: *p* < 0.05, dorsal striatum: *p* < 0.05, One–Way ANOVA with Holm-Sidak’s multiple comparisons test compared to whole-brain average expression).

To further explore the spatial distribution of Ctnnb1 transcription, we searched the *in-situ* hybridization (ISH) data from the Allen Brain Atlas (https://mouse.brain-map.org, experiment 196, 197, 79904258). Consistent with the high-throughput gene data from the bulk sample, the ISH data also showed high Ctnnb1 mRNA levels in the cortical subplate, hippocampus, and olfactory bulb. Moreover, higher Ctnnb1 transcription was also confirmed in the thalamus [the relay station of the brain (35) (Figures 2D,E, cortical subplate and thalamus: *p* < 0.001, hippocampus: *p* < 0.01, One–Way ANOVA with Holm-Sidak’s multiple comparisons test compared to whole-brain average expression)]. However, CTNNB1 transcription level was found average in the striatum and hypothalamus, but lower in the cerebellum (*p* < 0.01, One–Way ANOVA with Holm-Sidak’s multiple comparisons test compared to whole-brain average expression). Interestingly, the ISH data also provide information on layer-specific distribution, which showed significantly higher expression in the pyramidal and granule cell layer of CA1 and DG of the hippocampus and Purkinje layer of the cerebellum (Figure 2D).

Overall, both human and mouse data demonstrate relatively higher gene transcription of CTNNB1 or Ctnnb1 in the hippocampus, cerebellum, and thalamus in a layer-specific manner.

To investigate the significant role of CTNNB1 in the etiology and progression of NDDs, we conducted a comprehensive literature search in PubMed from 1/1/2002 to 11/31/2022, utilizing the following search terms: [(Ctnnb1) OR (CTNNB1) OR (β-catenin)] AND {[(NDD) OR (neurodevelopmental disorder)] OR [(ASD) OR (autism spectrum disorder)] OR [(ID) OR (intellectual disability)] OR [(SCZ) OR (schizophrenia)]}. Only English language publications were considered for inclusion in our review.

## 4. CTNNB1-related NDDs in human studies

Since the first discovery of loss-of-function mutations in ID patients, CTNNB1 mutation/loss of function had been found in different kinds of NDDs (36). A summary of recent publications related to the clinical phenotypes of patients with CTNNB1 mutation is presented in Table 1. Multiple case reports have described the complicated abnormalities caused by different mutations in CTNNB1, including *de novo* nonsense mutation (47), haploinsufficiency (42), small *de novo* deletions (51), with behavioral anomalies, anxiety, impaired motor performance, cognitive impairment as major phenotypes.

**Table 1:** Summary of recent CTNNB1-related NDDs in human studies.

Study	Study type	Mutation	Population	Main phenotype
(37)	Case report	Nonsense variation	1	NDD: Retinal detachment, lens and vitreous opacities, hypertonia of the extremities, mild thumb adduction, microcephaly, and developmental delay.
(38)	Case report	*De novo* mutation	1	NDD: Microcephaly, hypotonia, polydactyly, retinal detachment.
(39)	Case report	*De novo* mutation	1	NDD, microcephaly, and persistence of bilateral hyperplastic primary vitreous.
(40)	Case report	*De novo* variant	1	NDD with spastic diplegia and visual defects plus peripheral neuropathy.
(41)	Case–control	*De novo* mutations	Over 16,000	NDD
(13)	Case report	Loss-of-function mutations	13	NDD, spastic diplegia and visual defects.
(36)	Case–control	*De novo* mutation	100	ID
(42)	Case report	Haploinsufficiency	1	ID, truncal hypotonia, orofacial dyspraxia, hyperactivity, hyperopia, ataxic gait, and spasticity of the lower limbs.
(43)	Case report	*De novo* mutations	4	ID: progressive spastic diplegia, (primary) microcephaly, and significant additional craniofacial and brain abnormalities, including corpus callosum hypoplasia.
(44)	Case report	*De novo* mutation	16	ID
(45)	Case report	*De novo* nonsense mutation	1	ID, and microcephaly, exhibited hyperekplexia and apraxia of upward gaze.
(46)	Case report	*De novo* mutations	10	ID
(47)	Case report	Loss-of-function variants	24	ID, motor delay, speech impairment, dystonia and microcephaly.
(48)	Case report	*De novo* splice variant	1	Severe ID, ASD, microcephaly, absent or limited speech, facial dysmorphisms, peripheral hypertonia/spasticity, motor delay, and visual defects.
(49)	Case report	Loss-of-function mutation	1	ID, hypotonia, impaired vision, motor delay, and speech delay.
(50)	Case–control	*De novo* mutation	Over 14,597	ID and ASD
(51)	Case–control	*De novo mutation*	677	ASD
(52)	Case–control	Small *de novo* deletions	10,220	ASD, Febrile and afebrile seizures， head circumference deviation.
(53)	Case–control	*De novo* mutation	35,584	ASD
(54)	Case–control	*De novo* mutation	11,312	ASD
(55)	Case–control	*De novo* variants	46,612	ASD, developmental disorder
(56)	Case–control	Novel mutation	87	SCZ

ID, intellectual disability; ASD, autism spectrum disorder; SCZ, schizophrenia.

### 4.1. Non-specific NDDs

Patients with the CTNNB1 mutation have been found to exhibit NDDs-like characteristics. Four case reports have described the NDDs features of patients with CTNNB1 mutations: a patient (a 15-month-old boy) with a novel heterozygous nonsense variation (c.1627C > T, p.Gln558X) in exon 11 of the CTNNB1 displayed retinal detachment, lens and vitreous opacities, hypertonia of the extremities, mild thumb adduction, microcephaly, and developmental delay (37); another patient (a 15-month-old girl) with a *de novo* mutation (c.1603C > T, p.R535X) showed spastic diplegia, visual defects, and severe language problems (38); two additional patients with *de novo* CTNNB1 mutations (39) and/or variants (40) have been reported in 2022, presenting with microcephaly, persistent bilateral hyperplastic primary vitreous, and visual defects; 13 Korean patients, who were found to have CTNNB1 pathogenic loss-of-function variants (specifically c.1867C > T, p.Gln623Ter and c.1420C > T, p.Arg474Ter) manifested developmental delay and ID (13). CTNNB1 has further been identified as one of the high-risk NDDs genes in a large population (16,000) of NDDs cases, compared to nonpsychiatric controls, through large-scale targeted sequencing (41). The evidence suggests a potential association between mutations or nonsense variations in the CTNNB1 gene and NDDs.

Furthermore, multiple studies also clearly claimed the ID, ASD, or SCZ-like phenotypes caused by various CTNNB1 mutations.

### 4.2. ID

CTNNB1 was first identified as a potential candidate gene for ID in 2012, through diagnostic exome sequencing of 100 ID patients (36). Subsequent case reports have described ID patients with various types of CTNNB1 mutations: including a 3-year-old girl with a *de novo* 333 kb microdeletion who presented with developmental delay and postnatal microcephaly (42); 4 ID patients with CTNNB1 haploinsufficiency had abnormal craniofacial development (43); 16 ID patients with *de novo* heterozygous CTNNB1 mutations (44); an 11-year-old boy with a *de novo* mutation of CTNNB1 who presented with ataxia, truncal hypotonia, and speech impairment (45); an Iranian 8-year-old girl, with a loss-of-function mutation (c.1014G > A, p.Trp338Ter) in exon 7 of the CTNNB1 gene, exhibited a range of clinical manifestations including ID, hypotonia, impaired vision, motor delay, and speech delay (49). A study of 10 ID patients suggests that CTNNB1 mutations may be more prevalent in females, with 7 out of the 10 patients being female (46).

### 4.3. ID with ASD

Meanwhile, both ID and ASD-like features have been reported in patients with CTNNB1 mutations. For example, a case study reported a 32-year-old female patient with a *de novo* splice variant in CTNNB1 who presented with microcephaly, absent or limited speech, facial dysmorphisms, peripheral hypertonia/spasticity, motor delay, and visual defects (48). Additionally, 13 patients carrying the *de novo* truncating variant in CTNNB1 displayed impaired motor performance, ophthalmologic problems, and hand stereotypy (13). Furthermore, CTNNB1 has been identified as one of 208 candidate risk genes from a cohort comprising over 11,730 individuals with ID/ASD and 2,867 controls (50).

### 4.4. ASD

Nonsense and missense mutations of CTNNB1 have also been identified in individuals with ASD. In 2012, O’Roak et al. conducted exome sequencing on 677 individuals from 209 families and identified 126 risk genes with severe or disruptive *de novo* mutations, 39% of which were highly connected to the β-catenin/chromatin remodeling protein network, including CTNNB1 (51). Similarly, Sanders et al. reported the presence *of de novo* mutations in CTNNB1 in ASD patients (*n* = 10,220 from 2,591 families) in the Simons Simplex Collection data (52). The largest exome sequencing study of ASD to date (11,986 individuals with ASD, 23,598 control individuals) identified CTNNB1 as one of the 102 ASD-associated genes at a false discovery rate (FDR) of 0.1 or less (FDR_CTNNB1_ = 0.001 to 0.0001) (53). It has been further categorized into gene expression regulation groups and more frequently mutated in NDDs-ascertained cohorts. In the latest release of whole-genome sequencing (WGS) data from the Autism Speaks MSSNG resource (https://research.mss.ng, 5,100 individuals with ASD and 6,212 non-ASD parents and siblings), CTNNB1 (FDR 0.001 to 0.0001) was further identified as one of the 134 ASD-associated genes (54). Based on another single-cell nuclei transcriptomic data (55), 615 NDDs candidate genes (FDR < 0.05) had been identified and CTNNB1 was recognized as high-risk ASD genes in 46,612 trios by *de novo* enrichment analysis. CTNNB1 is identified as a category 1 gene (strong candidate ASD gene) according to the Simons Foundation Autism Research Initiative (SFARI) database.^1^

### 4.5. SCZ

Limited studies have reported CTNNB1 mutations in schizophrenia. In 2015, through sequencing genes related to the Wnt signaling pathway, Levchenko et al. found a novel CTNNB1 mutation (1943A > G, p.N648S) in 87 schizophrenia patients (56).

To summarize, all these case reports and sequencing studies from different patients suggest that lost-of-function mutations in CTNNB1 would cause a wide range of behavioral abnormalities related to NDDs, which could be further mainly characterized into ID, ASD, or SCZ.

## 5. Ctnnb1 related animal studies

**Figure 3:**
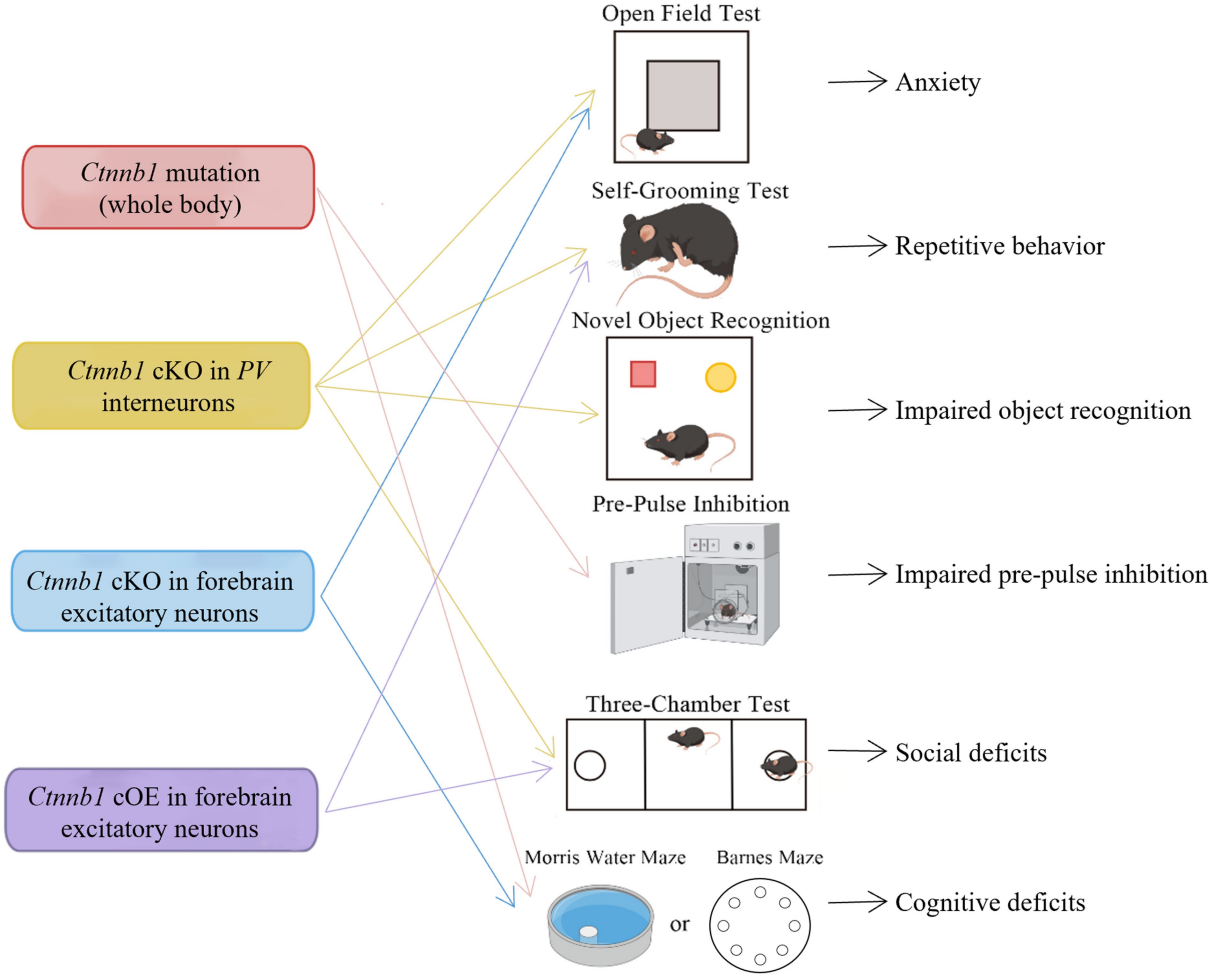
Behavioral deficits induced by Ctnnb1 loss of function or mutations in mice. Ctnnb1 conditional knockout (cKO) in parvalbumin (PV) interneurons induces anxiety, repetitive behavior, impaired object recognition, and social deficits. Ctnnb1 cKO in forebrain excitatory neurons causes repetitive behavior and cognitive impairment. Mice with whole-body Ctnnb1 mutations have impaired pre-pulse inhibition, repetitive behavior, as well as impaired social and cognition. Ctnnb1 cOE in excitatory neurons displayed repetitive behavior and social deficits.

**Figure 4:**
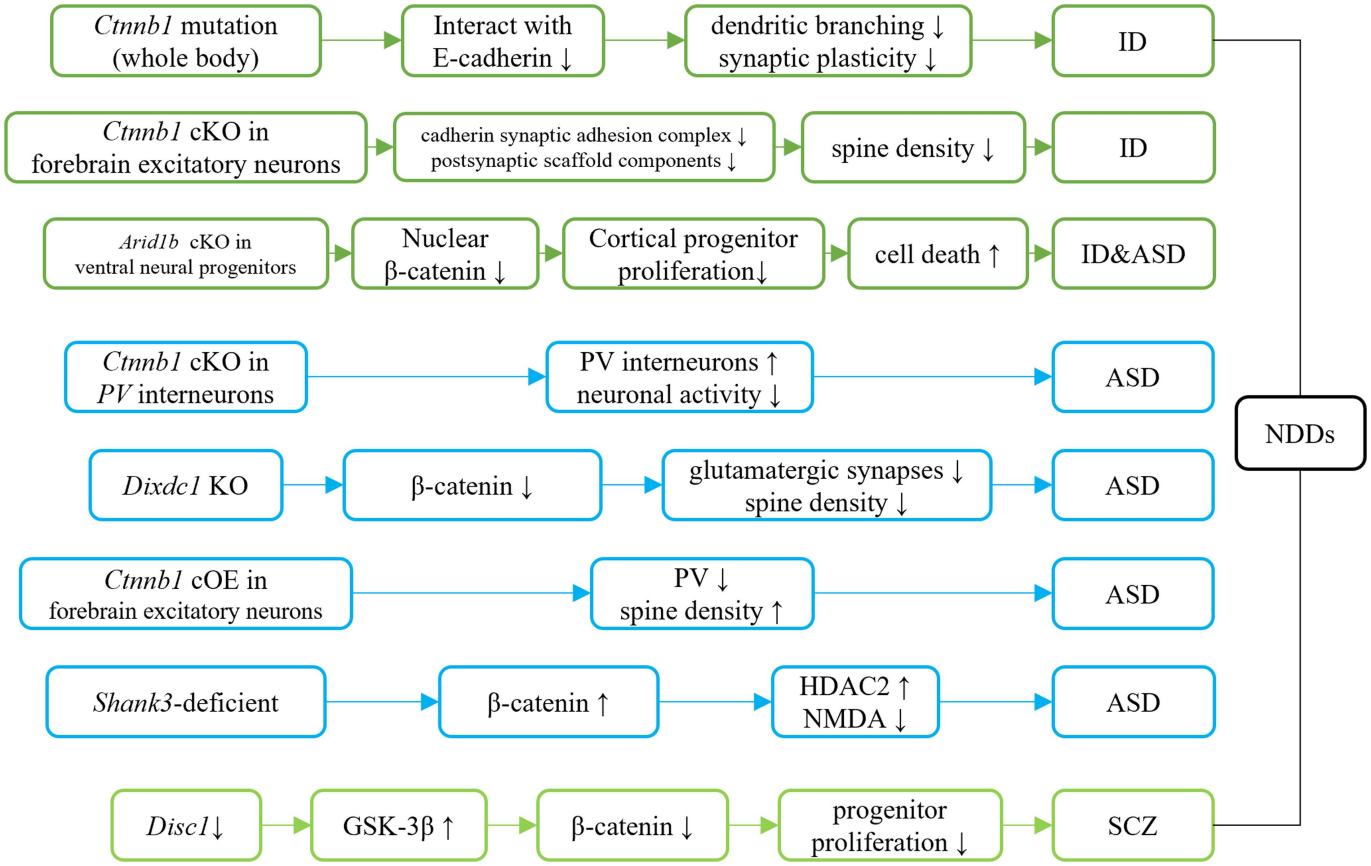
Ctnnb1 related signaling pathways in neurodevelopmental disorders. Dark green represents the direct/indirect link of Ctnnb1 to ID. Direct evidence of down-regulation of β-catenin suggests the β-catenin pathway involves ID. Indirect evidence indicates the Tcf4 mutation causes ID-like phenotypes in mice *via* down-regulation of β-catenin. Light blue represents the direct/indirect link of Ctnnb1 to ASD. Ctnnb1 deletion in PV interneuron results in social deficits in mice. Dixdc1 mutation and Shank3 mutation causes autism-like behaviors in mice *via* the suppressed Wnt/β-catenin signaling pathway. Disc1 deficiency impairs progenitor proliferation through the downregulation of β-catenin and introduces schizophrenia-like behaviors.

To gain a deeper understanding the roles of Ctnnb1 in NDDs, researchers have generated multiple mouse models with either insufficient or over-expressed levels of Ctnnb1, which has been summarized in Table 2, Figures 3, 4.

**Table 2:** Summary of Ctnnb1 deficiency and over-expression animal models.

Study	Tissue/ Cell type	Model	Method	Disease	Characteristics/Phenotype	Mechanisms
(43)	Whole body	Mice carry a Thr653Lys substitution in the C-terminal armadillo repeat β-catenin	Ctnnb1^Bfc/+^	ID	Deficits in cognitive function, sensorimotor gating, motor function, and disrupted ultrasonic vocalizations.	Deficits in dendritic branching and long-term potentiation.
(57)	Tissues derived from the neural tube	Ctnnb1 cKO mice	Brn4-Cre; β-cat^floxEx3–6/floxEx3–6^ mice	NDD	Reduced tissue mass of the spinal cord and brain.	Decreased progenitor pool.
(58)	Forebrain excitatory neurons	Ctnnb1 cKO mice	Ctnnb1^flox/flox^ x CaMKIIα-Cre mice	ID	Severe cognitive impairments and increased anxiety.	Reduced key synaptic adhesion and scaffold binding partners of β-catenin, reduced spine density.
(59)	PV interneurons	Ctnnb1 cKO mice	Ctnnb1^flox/flox^ x PV-Cre mice	ASD	Impaired novel object recognition, social novelty preference; increased anxiety, and repetitive behaviors.	Increased PV^+^ interneurons and reduced overall neuronal activity in the cortex.
(61)	Neural precursor cells	Ctnnb1 OE	∆90β-catenin transgenic mice	NDD	Enlarged brain.	Expanded precursor populations, increased neuronal production, cytoarchitectural distortions.
(57)	Tissues derived from the neural tube	Ctnnb1 cOE mice	Brn4-Cre; β-cat^+/floxEx3^ mice	NDD	Enlarged mass of the spinal cord and brain.	Increased neuronal precursor population.
(62)	Forebrain excitatory neurons	Ctnnb1 cOE	CaMKIIα-Cre; Ctnnb1^floxEx3/+^	ASD	Reduced social preference and novelty, increased repetitive behaviors.	Reduced parvalbumin but increased spine density.

cKO, conditional knockout; cOE, conditional over-expression.

### 5.1. Ctnnb1 deficiency animal models

#### 5.1.1. Whole body mutation

The Ctnnb1^Bfc/+^ mouse mutant (43), designated as “batface” (Bfc), exhibit abnormal craniofacial features due to a Thr653Lys substitution in the C-terminal armadillo repeat of β-catenin. This mutant also displayed ID-like behavior, including deficits in spatial learning and memory, fear conditioning, temporal cognition, and sensorimotor gating. Motor function and ultrasonic vocalizations were also disrupted in Bfc mutants (Table 2; Figure 3). The reduced affinity for membrane-associated cadherins, deficits in dendritic branching, and synaptic plasticity may contribute to the observed behavioral abnormalities (Figure 4).

#### 5.1.2. Whole body conditional knock out

Ctnnb1 conditional knock out (cKO) mice (Brn4-Cre; β-cat^floxEx3–6/floxEx3–6^) (57) with a targeted deletion in tissues derived from the neural tube were generated using the Cre-Loxp system. Reduced tissue mass in the spinal cord and brain was observed in this model, potentially due to a decrease in the progenitor pool for proliferation and an increase in cell death (Table 2).

#### 5.1.3. cKO in forebrain excitatory neurons

Mice with a Ctnnb1 cKO in forebrain excitatory neurons were generated by crossing Ctnnb1^flox/flox^ with CaMKIIα-Cre mice (58). Severe cognitive impairments, including deficits in spatial learning and memory, as well as increased anxiety were found in this model (Table 2; Figure 3). In addition to these behavioral phenotypes, reduced levels of key synaptic adhesion and scaffold binding partners of β-catenin were observed, along with reduced spine density (Figure 4).

#### 5.1.4. cKO in PV interneurons

The loss-of-function of Ctnnb1 in PV interneurons was generated through the crossing of Ctnnb1^flox/flox^ with PV-Cre mice (59). These mice exhibited ASD-like behaviors, including impaired novel object recognition, reduced social novelty preference, increased repetitive behaviors, and anxiety (Table 2; Figure 3). Significantly reduced overall neuronal activity was also observed in the cortex of these cKO mice, accompanied by an increase in PV^+^ interneurons. These findings suggest that Ctnnb1 may play an important role in maintaining the excitatory/inhibitory balance (Figure 4).

#### 5.1.5. Other models with indirect down-regulation of β-catenin

Instead of a mutation or knockout of Ctnnb1, several transgenic animal lines also exhibit indirect downregulation of β-catenin.

The GSK-3β/β-catenin signaling pathway was disrupted in a mouse model of schizophrenia with a loss of function of DISC1 (60). This disruption was characterized by the over-expression of GSK-3β and suppression of β-catenin. The mice exhibited behavioral deficits, including hyperlocomotion and depression-like behaviors (as demonstrated by increased immobility in the forced swimming test). These deficits were likely due to the impairment of progenitor proliferation caused by DISC1 knockdown (Figure 4). However, stable expression of β-catenin was able to override the defects in progenitor proliferation and rescue behavioral abnormalities in these mice.

Suppressed β-catenin has also been found in mice lacking DIX domain containing-1 (DIXDC1) (63), a protein involved in the intracellular Wnt/β-catenin signaling pathway, which exhibit abnormal anxiety, depression, and social deficits. Pyramidal neurons in the brains of these animals had reduced dendritic spines and glutamatergic synapses (Figure 4). Treatment with lithium or a GSK-3β inhibitor showed to correct the behavioral and neurodevelopmental phenotypes in these animals.

Decreased nucleus localization of β-catenin was found in mice with Arid1b cKO in ventral neural progenitors (Dlx-Cre; Arid1b^flox/flox^) (64), that displayed pronounced ID and ASD-like behaviors. Decreased proliferation of cortical and ventral neural progenitors, as well as altered cell cycle regulation and increased cell death, were also observed in this animal model (Figure 4).

### 5.2. Ctnnb1 over-expression animal models

Additionally, animal models with over-expression of Ctnnb1 have also been developed.

#### 5.2.1. Whole body conditional over-expression

The Brn4-Cre; β-cat^+/floxEx3^ mice (57) were generated with conditional over-expression (cOE) of Ctnnb1. These mice with gain-of-function in β-catenin exhibited an enlarged mass of the spinal cord and brain, which may cause by an increased neuronal precursor population (Table 2).

#### 5.2.2. cOE in forebrain excitatory neurons

Mice with cOE of β-catenin in forebrain excitatory neurons (CamKIIα-Cre; Ctnnb1^floxEx3/+^) (62) demonstrated reduced social and social novelty preference, as well as increased repetitive behaviors, such as repetitive circling (Table 2; Figure 3). Next-generation sequencing of RNA revealed dysregulation of several genes, including those involved in neuron projection development, neuron differentiation, canonical Wnt target genes, as well as several genes that have been implicated in human ASD. The underlying mechanisms are likely due to reduced parvalbumin at both the mRNA and protein levels, as well as increased dendritic spine density (Figure 4).

#### 5.2.3. Other models with indirect up-regulation of β-catenin

In a widely utilized animal model of ASD, Shank3-deficient mice, both nucleus and cytosolic β-catenin were found to be upregulated (65). These mice also exhibited high expression of HDAC2 and suppressed NMDA function (Figure 4). Downregulation of β-catenin *via* β-catenin shRNA resulted in improved social preference in these mice.

The above findings suggest that disruptions in β-catenin expression, whether suppressed or overexpressed, can lead to abnormalities in neuronal proliferation and differentiation, which in turn contribute to the pathogenesis of NDDs such as ID, ASD, and SCZ. These findings highlight the importance of β-catenin in normal neurodevelopment and underscore the potential therapeutic value of targeting this protein in the treatment of NDDs.

## 6. Potential treatment in NDDs by targeting Wnt/β-catenin signaling pathway

As the canonical Wnt/β-catenin pathway is one of the major pathways involve in NDDs (66), targeting this signaling pathway would provide potential treatment for NDDs. For the following part, we continue summarizing different kinds of drugs that target the pathogenesis of NDDs due to dysregulation of the Wnt/β-catenin pathway. Several drugs have been identified potential treatment in NDDs by regulating β-catenin directly or indirectly.

### 6.1. Lithium

Lithium is a widely used drug that targets the Wnt/β-catenin signaling pathway to treat psychiatric disorders. As a classic mood stabilizer, lithium inhibits GSK-3β, a key enzyme in the Wnt signaling pathway, and up-regulates β-catenin (67). Although originally used to treat mania due to its tranquilizing effects (68), several studies have explored the potential therapeutic use of lithium in NDDs. For example, lithium treatment in ASD patients with SHANK3 mutations has been shown to improve behavioral symptoms and allow patients to recover their pre-catatonia level of functioning, with limited side effects (69).

In a Fragile X mouse model, lithium down-regulated GSK-3β activity and reduced the audiogenic seizures (70). Treatment with lithium rescued the behavioral performance in mice with Tbr1 haploinsufficiency, another high-risk gene for ASD, which lasts over 6 months. Chronic lithium treatment has also been shown to enhance spatial learning and memory (71), as well as to have anti-aggression (68) and anti-depression effects (72). However, it is important to note that prolonged maintenance of high serum lithium concentrations can lead to kidney disease (73).

### 6.2. SB216763

SB216763 is a highly potent and selective GSK-3β inhibitor with an IC50 of 0.2 μM and 96% inhibition at 10 μM. It has been successfully utilized to boost β-catenin by inhibiting GSK-3β in the treatment of various animal models with NDDs. For instance, the administration of 2 mg/kg SB216763 can rescue SCZ-like behavioral deficits caused by Disc1 KO (60). The same dose also proved to be effective in treating hippocampus-dependent learning deficits in Fmr1 KO mice (74). Furthermore, 10 mg/kg SB216763 administration has been shown to normalize the ASD-like behavioral outcomes in Dixdc1 KO (63). SB-216763 has been shown to effectively reduce audiogenic seizures and normalize open field behavior in Fragile X mice when administered at a dosage of 4–10 mg/kg (70).

### 6.3. Sulindac

As an FDA-approved anti-inflammatory drug, sulindac is another commonly used drug that serves as a β-catenin inhibitor (75). Sulindac has been shown to significantly inhibit β-catenin transcription in cancer cell studies (76). It has also been evaluated in valproic acid (VPA) induced ASD models and has been found to reduce VPA-induced stereotypic-like activity, anxiety (77), and other autism-like phenotypes in rats (78, 79). In cell culture studies, it has been demonstrated that Sulindac increases cell viability and reducing oxidative stress in VPA-treated primary neurons *via* suppressing β-catenin mRNA level (77). These findings suggest that small-molecule targeting of the Wnt/β-catenin pathway, such as sulindac, may be a promising approach for improving behavioral phenotypes in individuals with ASD.

### 6.4. PPARγ agonist

Another drug targeting to Wnt/β-catenin signaling pathway in NDDs would be a peroxisome proliferator-activated receptor gamma (PPARγ) agonist. PPARγ is a ligand-activated transcriptional factor, which interacts with other coactivator proteins to induce specific gene expression (80). In the Wnt/β-catenin pathway, PPARγ interacts with TCF/LEF domain and the β-catenin-binding domain (81). As an agonist of PPAR, pioglitazone showed anti-inflammation effects on ASD patients, which alleviated ASD-like behavior, such as irritability, lethargy, stereotypy, and hyperactivity (82). In the lipopolysaccharide-induced ASD rat model, pioglitazone treatment exhibited enhancement in social interaction and vocalization (83). Meanwhile, in a maternal immune SCZ rat model, pioglitazone, also mitigated the male offspring’s schizophrenia-like behaviors when given before parturition (84).

According to these clinical and preclinical studies, targeting the Wnt/β-catenin signaling pathway with lithium, SB216763 and sulindac, or targeting the transcriptional factor with PPARγ agonist may have therapeutic effects for NDDs.

## 7. Conclusion and future perspectives

Over the past decade, our understanding of the role of CTNNB1 and the Wnt/β-catenin signaling pathway in brain development has greatly increased following the identification of CTNNB1 as a risk gene for NDDs. Within the somatic system, CTNNB1 was initially identified as a potential tumor suppressor gene. Mutations in this gene can cause disruptions in the Wnt signaling pathway, leading to the formation of tumors in a variety of cancers (85, 86). Furthermore, mutations in CTNNB1 can result in decreased cell adhesion and epithelial to mesenchymal transition, allowing tumor cells to migrate and metastasize (87). However, within the central nervous system, CTNNB1 mutations can lead to defects in synaptic localization and stabilization, which can cause an imbalance between excitatory and inhibitory signaling, ultimately resulting in NDDs (88, 89). These findings suggest that CTNNB1 may have distinct roles within the somatic system and central nervous system, highlighting the need for further research in this area.

Studies have demonstrated the crucial role of CTNNB1 in central nervous system development. Individuals with CTNNB1 mutations exhibit NDDs-associated phenotypes. Moreover, various animal models with Ctnnb1 deletion/*de novo* mutations have been generated to replicate the core clinical features observed in NDDs patients. Several reagents and targets have been identified as potential treatments for diseases related to CTNNB1 mutations.

Based on high-throughput data from the human brain, it is known that CTNNB1 is highly expressed during embryonic stages and early childhood, suggesting that CTNNB1 plays an important role in brain development. Current transgenic animal models have covered all of these stages. It would be of scientific interest to examine the biological characteristics of high CTNNB1 expression during adolescence, as this would provide valuable insights into the role of CTNNB1 in postnatal psychiatric disorders.

As summarized previously, Ctnnb1 has been identified as highly expressed in the cerebellum, hippocampus, thalamus, and cerebral cortex. However, the specific effects of Ctnnb1 in these brain regions remain unknown, as previous studies have focused on the whole body or whole brain mutation of Ctnnb1. To further understand the function of Ctnnb1 in these high-expression regions, studies using local AAV infusion into the target region are necessary. In addition, ISH data suggest that Ctnnb1 expression exhibits a specific spatial distribution, including extremely high expression in the pyramidal and granule cell layers of the CA1 and DG of the hippocampus, and the Purkinje layer of the cerebellum. ISH data also indicate layer differences within the PFC. Advanced spatial transcriptome technologies such as MERFISH (90) and stereo-seq (91) could provide valuable information on the function of Ctnnb1 in different layers. Furthermore, the use of multi-patch techniques would be beneficial in exploring the microcircuit changes.

Interestingly, variable levels of Ctnnb1 expression were observed in the cerebellum using different techniques. High-throughput sequencing of mRNA extracted from brain tissue was conducted to determine the relative mRNA expression of Ctnnb1, as presented in Figures 2B,C. This method is prone to affected by cell numbers. And since the cerebellum possesses a considerably higher number of cells, especially in the granule layer, the average expression level is relatedly high. However, *in situ* RNA staining was performed in Figures 2D,E to determine the spatial distribution of Ctnnb1 in brain slices. The fluorescence intensity is indicative of Ctnnb1 expression levels and may be influenced by the total volume of the specific brain region. Despite the high expression of Ctnnb1 in the granule layer of the cerebellum, the average expression level was relatively low due to the lower Ctnnb1 expression in the molecular layer of the cerebellum.

With the development of various transgenic animals, scientists have studied the effects of knocking out the Ctnnb1 in specific cell types using the Cre-Loxp system. A previous study demonstrated that cKO of Ctnnb1 in forebrain excitatory neurons leads to severe cognitive impairments (58), indicating a significant role of Ctnnb1 in pyramidal neurons. However, there is a lack of research on its function in other types of excitatory neurons, such as VGLUT2, which is a primary cell type in the thalamus (92). Additionally, cKO of Ctnnb1 in PV interneurons has been found to result in ASD-like symptoms (59), further suggesting a crucial role for Ctnnb1 in interneurons. However, the function of Ctnnb1 in other types of interneurons, such as VIP and SST interneurons, remains to be further explored. Furthermore, the function of Ctnnb1 in non-neuronal cells, including astrocytes, glia, and microglia within the brain, has not been reported and warrants further investigation.

To date, most researches have been focused on characterizing the behavioral phenotypes of different Ctnnb1 KO animal models. However, the underlying mechanisms, particularly neuroanatomical and functional changes, have not been fully investigated. Electrophysiological studies, such as Patch-seq, could provide further evidence in this regard. Additionally, long-range circuit studies have been lacking. The use of optogenetics and chemogenetics could allow for exploration in cognitive or ASD-related circuits, such as within the hippocampal CA3-CA1, cortical-striatal, cortical-thalamic, and thalamic-striatal.

In the aspects of treatment, several drugs have been proposed with therapeutic effects for NDDs. These drugs either directly modulate β-catenin function (LiCl and sulforaphane) or indirectly modulate its expression (PPARγ). Future research utilizing proteomics, RNA-seq, and ATAC-seq may help to identify additional downstream molecular targets of β-catenin and epigenetic targets for Ctnnb1 transcription.

Overall, in-depth studies are required to investigate the impact of Ctnnb1 mutations in a specific manner by brain region, cell type, and layer. Neuroanatomical and functional neural activity and transmission studies should be conducted to understand the effects on micro-circuits and long-distance circuits. New high-throughput techniques such as proteomics, RNA-seq, and ATAC-seq could be utilized to identify potential therapeutic targets for Ctnnb1-induced NDDs.

## Author contributions

WZ conducted the literature search and drafted the manuscript. WW and TY wrote part of the manuscript. WS and TT designed the outline of the review, supervised the entire process, and revised the manuscript. All authors contributed to the article and approved the submitted version.

## Funding

This work was supported by funding to WS from Oujiang Laboratory (OJQDJQ2022002), to TT from Oujiang Laboratory (OJQD2022002).

## Conflict of interest

The authors declare that the research was conducted in the absence of any commercial or financial relationships that could be construed as a potential conflict of interest.

## Publisher’s note

All claims expressed in this article are solely those of the authors and do not necessarily represent those of their affiliated organizations, or those of the publisher, the editors and the reviewers. Any product that may be evaluated in this article, or claim that may be made by its manufacturer, is not guaranteed or endorsed by the publisher.

## Footnotes

^1^https://gene.sfari.org
